# Supplementary material for: Household costs associated with zoonotic Plasmodium knowlesi, P. falciparum, P. vivax and P. malariae infections in Sabah, Malaysia
Source: PLoS Negl Trop Dis. 2025 Apr 4;19(4):e0012180. doi: 10.1371/journal.pntd.0012180 (PMC11970689; doi:10.1371/journal.pntd.0012180)
Supplement: S1 Text — (DOCX) [file pntd.0012180.s001.docx]

# **S1 Text**

**Table A: Mean number of days travelled (standard deviation) for medical care per episode of malaria (n=152).**

|  | *P. vivax* (n=22) | *P. falciparum* (n=16) | *P. malariae* (n=6) | Non-severe *P. knowlesi* (n=101) | Severe *P. knowlesi* (n=7) | Total |
| --- | --- | --- | --- | --- | --- | --- |
| Before hospital admission | 0.2 (0.8) | 1.4 (1.3) | 1.8 (2.6) | 1.4 (1.8) | 0.4 (0.5) | 1.2 (1.7) |
| Since hospital admission | 0.6 (1.2) | 3.0 (1.7) | 2.0 (1.3) | 1.5 (1.8) | 0.9 (1.2) | 1.5 (1.8) |
| Total travel days | **0.8 (1.8)** | **4.4 (2.3)** | **3.8 (3.0)** | **2.9 (3.3)** | **1.3 (1.7)** | **2.7 (3.1)** |

### **Table B: Total mean and median household costs for a single malaria episode in 2023 Malaysian ringgit (n=152).**

|  | Mean | Standard deviation | Percent of total cost | Median | Interquartile range |
| --- | --- | --- | --- | --- | --- |
|  |  |  |  |  |  |
| Hospital admission | 25.79 | 40.07 | 20% | 12.31 | 4.96 – 34.78 |
| Clinic visit | 13.83 | 23.79 | 11% | 7.46 | 4.92 – 12.42 |
| Medicine | 2.58 | 11.12 | 2% | 0.00 | 0.00 – 0.90 |
| Travel to clinic and hospital | 10.33 | 17.36 | 8% | 4.97 | 0.00 – 12.42 |
| *Total direct costs* | 52.53 | 64.53 | 41% | 31.42 | 19.92 – 54.29 |
|  |  |  |  |  |  |
| Patient time | 52.25 | 57.29 | 41% | 35.83 | 13.79 – 64.49 |
| Caregiver time | 21.68 | 27.68 | 17% | 17.35 | 7.16 – 25.96 |
| *Total indirect costs* | 73.93 | 67.91 | 58% | 54.15 | 29.58 – 93.15 |
|  |  |  |  |  |  |
| *Total other costs^1^* | 0.78 | 5.77 | 0.6% | 0.00 | 0.00 – 0.00 |
|  |  |  |  |  |  |
| Total costs | 127.24 | 99.66 | 100% | 93.69 | 68.95 – 149.15 |

^1^Other costs were unspecified in the questionnaire; no details about the nature of these costs were provided by the participants.

**Table C: Total mean and median household costs for a single malaria episode by sex in 2023 United States dollars (n=152)**

|  | Male (n=114) | | | | Females (n=38) | | |  | | |  |
| --- | --- | --- | --- | --- | --- | --- | --- | --- | --- | --- | --- |
|  | **Mean** | **Standard deviation** | **Median** | **Inter-quartile Range** | **Mean** | **Standard deviation** | **Median** | | **Inter-quartile range** | **P-value (Mann Whitney test)** | |
| Hospital admission | 23.51 | 32.71 | 12.75 | 5.09 – 25.49 | 35.26 | 59.37 | 12.53 | | 0.00 – 15.03 | 0.841 | |
| Clinic visit | 13.02 | 23.51 | 7.51 | 5.01 – 12.75 | 17.73 | 26.94 | 10.11 | | 6.19 – 15.29 | 0.068 | |
| Medicine | 2.87 | 12.71 | 0.00 | 0.00 – 1.28 | 2.01 | 6.17 | 0.00 | | 0.00 – 0.00 | 0.083 | |
| Travel to clinic and hospital | 9.04 | 15.54 | 5.10 | 0.00 – 12.53 | 15.26 | 22.95 | 5.13 | | 0.00 – 12.90 | 0.455 | |
| Total direct costs | 48.44 | 56.02 | 30.71 | 20.39 – 51.24 | 70.26 | 89.07 | 40.17 | | 22.55 – 84.12 | 0.018 | |
| Time off work | 62.69 | 64.32 | 47.93 | 18.88 – 88.21 | 26.34 | 21.04 | 29.41 | | 0.00 – 36.76 | 0.018 | |
| Caregiver time | 22.69 | 31.03 | 17.26 | 5.31 – 24.71 | 20.87 | 18.70 | 18.97 | | 9.43 – 26.86 | 0.856 | |
| Total indirect costs | 85.38 | 76.18 | 63.84 | 32.62 – 113.13 | 47.22 | 30.98 | 41.28 | | 23.87 – 58.81 | 0.010 | |
| Other costs | 0.44 | 4.75 | 0.00 | 0.00 – 0.00 | 1.86 | 8.51 | 0.00 | | 0.00 - 0.00 | 0.020 | |
| Total costs | 134.27 | 103.18 | 96.77 | 71.65 – 156.38 | 119.34 | 99.86 | 88.40 | | 63.06 – 141.92 | 0.329 | |

**Table D: Total mean, standard deviation (SD), median, and interquartile (IQR) ranges for household costs per malaria episode for working-age male adults (aged 16-65) as compared to the rest of the population in 2023 United States dollars (n=152). P-values were calculated using Mann Whitney tests.**

|  | Working aged males (n=82) | | | | Everyone else (n=70) | | | |  |
| --- | --- | --- | --- | --- | --- | --- | --- | --- | --- |
|  | **Mean** | **SD** | **Median** | **IQR** | **Mean** | **SD** | **Median** | **IQR** | **P-value** |
| Hospital admission | 22.04 | 34.26 | 12.53 | 5.09 – 25.49 | 31.64 | 47.65 | 12.75 | 5.01 – 53.88 | 0.404 |
| Clinic visit | 14.59 | 27.32 | 7.65 | 4.13 – 12.75 | 13.73 | 20.67 | 8.38 | 5.09 – 13.47 | 0.331 |
| Medicine | 3.42 | 14.79 | 0.00 | 0.00 – 1.29 | 1.75 | 5.18 | 0.00 | 0.00 – 0.00 | 0.006 |
| Travel to clinic and hospital | 10.02 | 17.70 | 5.13 | 0.00 – 12.53 | 11.27 | 18.03 | 5.09 | 0.00 – 12.75 | 0.865 |
| Total direct costs | 50.06 | 62.89 | 29.82 | 20.39 – 50.98 | 58.38 | 70.06 | 40.17 | 20.64 – 73.92 | 0.204 |
| Time off work | 76.27 | 59.68 | 61.62 | 29.41 – 99.68 | 27.04 | 45.29 | 19.01 | 0.00 - 36.76 | <0.001 |
| Caregiver time | 19.39 | 27.93 | 14.70 | 0.00 – 22.06 | 25.58 | 28.78 | 20.25 | 14.02 – 29.41 | 0.003 |
| Total indirect costs | 95.66 | 73.94 | 73.51 | 44.23 – 117.95 | 52.62 | 56.47 | 37.85 | 20.91 – 58.81 | 0.000 |
| Other costs | 0.62 | 5.61 | 0.00 | 0.00 – 0.00 | 1.01 | 6.30 | 0.00 | 0.00 – 0.00 | 0.243 |
| Total costs | 146.34 | 106.90 | 109.65 | 77.40 – 161.23 | 112.01 | 93.88 | 82.74 | 60.85 – 131.25 | 0.006 |

**Table E: Total mean household costs per malaria episode by species in 2023 United States dollars (n=152).**

|  | *P. knowlesi* (n=108) | | *P. vivax* (n=22) | | *P. falciparum* (n=16) | | *P. malariae* (n=6) | |
| --- | --- | --- | --- | --- | --- | --- | --- | --- |
|  | **Mean** | **Standard deviation** | **Mean** | **Standard deviation** | **Mean** | **Standard deviation** | **Mean** | **Standard deviation** |
| Hospital admission | 21.87 | 26.37 | 56.04 | 84.16 | 21.25 | 22.56 | 14.38 | 18.17 |
| Clinic visit | 9.85 | 9.12 | 36.42 | 52.50 | 15.34 | 25.59 | 7.80 | 6.14 |
| Medicine | 3.45 | 13.35 | 1.10 | 4.01 | 0.08 | 0.15 | 0.65 | 1.10 |
| Travel to clinic and hospital | 10.20 | 9.34 | 15.77 | 24.96 | 5.36 | 7.02 | 12.63 | 9.34 |
| Total direct costs | 45.38 | 44.54 | 109.33 | 127.98 | 42.04 | 37.01 | 35.45 | 32.08 |
| Time off work | 52.59 | 55.33 | 56.73 | 80.47 | 57.75 | 57.06 | 49.32 | 41.02 |
| Caregiver time | 20.84 | 24.13 | 32.98 | 43.97 | 19.90 | 31.64 | 14.20 | 11.32 |
| Total indirect costs | 73.43 | 65.50 | 89.72 | 88.20 | 77.65 | 79.65 | 63.53 | 43.44 |
| Other costs | 1.13 | 7.01 | 0.00 | - | 0.00 | - | 0.00 | - |
| Total costs | 119.93 | 81.39 | 199.05 | 173.87 | 119.68 | 82.68 | 98.98 | 41.85 |

**Table F: Marginal costs, standard errors and 95% confidence intervals of factors associated with variability with total household costs from the generalized linear model that only includes *P. knowlesi* patients (N=108).** All costs are in 2023 United States dollars.

|  | Marginal cost from base case (dy/dx) | Standard error | P-value | 95% confidence interval |
| --- | --- | --- | --- | --- |
| Sex (male) | 25.68 | 18.41 | 0.163 | -10.41, 61.76 |
| Age | 1.11 | 0.47 | 0.019 | 0.18, 2.03 |
| Severe malaria | 61.39 | 33.53 | 0.067 | -4.34, 127.11 |
| Anemia | 6.25 | 17.79 | 0.725 | -28.60, 41.12 |

**Table G: Generalized linear model of all malaria patients with log link output of coefficients, standard errors and 95% confidence intervals of covariates associated with total household costs.** All costs are in 2023 United States dollars.

|  | Coefficient | Standard Error | P-value | 95% Confidence Interval |
| --- | --- | --- | --- | --- |
| Severe malaria | 0.446 | 0.301 | 0.138 | -0.14, 1.04 |
| Sex (male) | 0.143 | 0.142 | 0.314 | -0.14, 0.40 |
| Age | 0.011 | 0.004 | 0.003 | 0.00, 0.02 |
| Anemia | 0.119 | 0.143 | 0.404 | -0.16, 0.40 |
| Human-only malaria | 0.384 | 0.132 | 0.004 | 0.12, 0.64 |
| Cons | 4.15 | 0.228 | <0.001 | 3.70, 4.60 |

Log likelihood = **-**880.74; Akaike Information Criterion = 11.745; Bayesian Information Criterion = **-**663.277

**Fig A: Total household costs per malaria episode by age for the scenario analyses in 2023 United States dollars.** The base case uses reported incomes with mean income applied to all adults who did not report income. Scenario 1 uses only reported incomes to value productivity losses. Scenario 2 applies the Malaysian Household Income Survey wage [1] to adults. Scenario 3 applies the Malaysian Household Income Survey wage [1] to all patients including children. Scenario 4 applies the Household Inequities Survey wage [2] to adults. Scenario 5 applies the Household Inequities Survey wage [2] to all patients including children.

**References**

1. Department of Statistics M. Household Income Survey Putrajaya, Malaysia: Department of Statistics Malaysia; 2012 [cited 2022 July 15th 2022]. Available from: https://www.dosm.gov.my/v1/index.php?r=column/cthemeByCat&cat=120&bul_id=d1FLak9XZklSMlVqZkhoZUloZytYQT09&menu_id=amVoWU54UTl0a21NWmdhMjFMMWcyZz09.

2. Tey NP, Lai SL, Ng ST, Goh KL, Osman AF. Income inequality across states in Malaysia. Planning Malaysia. 2019;17.
