# Supplementary material for: Household costs associated with zoonotic Plasmodium knowlesi, P. falciparum, P. vivax and P. malariae infections in Sabah, Malaysia
Source: PLoS Negl Trop Dis. 2025 Apr 4;19(4):e0012180. doi: 10.1371/journal.pntd.0012180 (PMC11970689; doi:10.1371/journal.pntd.0012180)
Supplement: Attachment — Submitted filename: Malaria costs in Malaysia review.docx [file pntd.0012180.s002.docx]

**Review**

**The economic burden of zoonotic Plasmodium knowlesi malaria on households in Sabah, Malaysia compared to malaria from human-only Plasmodium species**

**PNTD-D-24-00626**

Thank you for the opportunity to review this paper. I have no major methodological concerns. My feedback is mainly on the framing of the study and flow of the paper.

1. In my view, the most interesting/important finding was that most patients are incurring catastrophic health expenditures regardless (if I’m understanding it correctly) of the species of malaria infection and despite the country’s policy of covering malaria treatment expenses. I don’t know the Malaysian context well, but I suspect that if malaria elimination is the goal, there is already motivation to address *P. knowlesi*. The fact that P. knowlesi costs are not very different from other malaria costs may just reinforce the status quo. It could be a more impactful paper if it has a health financing message rather than a malaria control message.
2. If you opt to keep the current framing, the Background section needs to make a stronger case as to why comparing the costs of different species of malaria is important, and better justify the need for the subgroup analysis. Including hypotheses would be helpful. What cost differences did you expect there to be, and why?

Some of this content could be relocated from the Discussion, which is quite long. There is also some contextualization in the Results (lines 256-7), which should be moved elsewhere.

3. This is a small concern, but under “Ethics,” it would be better to list all of the IRB’s than to say the IRB’s included XYZ.
4. I found the “study design and participants” section difficult to follow. Considering separate paragraphs for “study design” and “participants.”
5. Because the species of malaria infection correlates with patient characteristics (e.g. people with *P. knowlesi* are more likely to be men), I’m left wondering whether it is the type of malaria infection or the characteristics of the patients driving these differences in cost. Are you able to disentangle that at all?
