# Supplementary material for: Household costs associated with zoonotic Plasmodium knowlesi, P. falciparum, P. vivax and P. malariae infections in Sabah, Malaysia
Source: PLoS Negl Trop Dis. 2025 Apr 4;19(4):e0012180. doi: 10.1371/journal.pntd.0012180 (PMC11970689; doi:10.1371/journal.pntd.0012180)
Supplement: Attachment — Submitted filename: Response to Reviewers.docx [file pntd.0012180.s004.docx]

We thank you for the detailed, insightful comments provided and believe they have improved the manuscripts quality and messaging. This letter details the changes we have made to the manuscript.

**Additional Editor Comments:**

The text p15 on subgroups has some statistical errors. L232 “statistically insignificant” – do you mean non-significant? ‘Insignificant’ would change the meaning and imply that it is not important, non-significant would convey that p>0.05. L244 “p=0.000” – this p-value cannot be zero, although it can be given as 0.00 in the software output due to rounding. It is usually written for example ‘p<0.001.’ L231: It is misleading to state that “there are differences but they are not significant”. There is simply no evidence of a difference and the difference that you observe is likely to have arisen by chance. Please be careful of wording for statistical concepts throughout the manuscript.

We have changed the wording on line 243, to say “non-significant.” We have also updated the p-value on line 255 to state “p<0.001”.

We have also changed the wording of line 231 to ensure the statement isn’t misleading, to

“Male participants reported a slightly greater average cost of illness with US$134.26 compared to US$119.35 for females; however, this difference was statistically non-significant (Table 4).”

We have further analyzed the remainder of the manuscript for consistency and have not found any further examples of this.

The sample size is very small for some of the malaria species (n=6 for P. malariae, n=16 for P. falciparum, n=22 for P. vivax, n=108 for P. knowlesi). This could be more prominently acknowledged and borne in mind while interpreting the results.

Thank you for this comment. We have acknowledged this in the following sections:

- Discussion, lines 333-335 “Malaysia has successfully controlled human-only malaria in recent years, with only small numbers of imported cases of *P. vivax* and *P. falciparum* now reported [1].”
- Discussion, lines 366-368 “To our knowledge, no previous studies have shown the cost of *P. vivax* infections to be higher than *P. falciparum* infections, however, this should be interpreted cautiously due to the low case numbers of *P. falciparum*.”
- Limitations, lines 460-462: “Lastly, the data in this study is dated, with no indigenous cases of P. *falciparum* and *P. vivax* present in the area, which limits the generalizability of these findings.”

Did you include all patients presenting between February 2013 to September 2016? If not, how were they selected?

All sequential malaria cases managed at these hospitals were invited to participate, and we have changed the wording within the study design and participants section of the methods, lines 116-119:

“A pre-tested, structured, closed-ended questionnaire [26] was directly administered to all patients at clinics by trained research nurses to malaria patients or caregivers at around 28 days after hospital admission around 14 to 28 days after hospital admission to estimate the costs of a single malaria episode [12]”

It is not obvious to see how you can disentangle all the factors with this sample size. As one reviewer mentions, the male and female differences observed could be due to residual confounding. I suspect you are making more of this than is justified by the data.

We appreciate this comment and concern. We have attempted to disentangle these results through statistical analysis and the two iterations of the Generalized Linear Model. The main findings of the study are the overall costs to households, and the novelty of having cost estimates from four species of malaria in the same setting, rather than cost differences which may have or have not arisen from sex. We also note that sex was statistically non-significant both using Mann-Whitney tests, and within the regression model when considering other variables. We have emphasized more important findings throughout the results, discussion and abstract, by only highlighting justified findings. Additionally, we have stated in the discussion:

“Without further data, it is challenging to speculate on other factors associated with higher total costs per malaria episode. However, when controlling for malaria severity, malaria from human-only *Plasmodium* species (predominantly influenced by higher *P. vivax* related costs) was associated with higher total costs (Table 5), which may be reflective of the longer duration of illness with *P. vivax* and *P. falciparum* compared with non-severe *P. knowlesi* (Table 2).”

The patients came from three hospitals – the costs may vary between hospitals (and this may cause a lack of statistical independence between observations), however hospital does not seem to have been taken into account in the analysis.

We have conducted a further analysis and stratified the results in Table 4 by hospital setting; however, these rural district hospitals are all adjacent and based in similar socio-economic settings.

There are many p-values in this manuscript. Doing many tests can lead to the problem of multiple comparisons. For one hypothesis test, if there is no difference (the null hypothesis is true), there is still a 5% chance of a false positive. If you have do many tests, then the risk of at least one false positive increases. Did you consider this?

We did consider the number of p-values and statistical tests we performed during this analysis and attempted to ensure that we only reported major findings with credible effect sizes and high confidence. Additionally, the two regression models (GLMs) we performed control for potential confounders and add to the robustness of our results. Many of the p-values we reported were statistically significant not only at a threshold of p<0.05, but 0.01 and 0.001, and we have now stated these throughout the manuscript and report associated effect sizes for comparative analyses. The narrative of the findings presented does not focus on the results of any single p-value, thus if there are any false positives as you have suggested, the overall message of the analysis should not be heavily impacted.

Please give a measure of uncertainty (eg SD, 95% CI etc) when giving point estimates (eg a mean, a measure of the strength of an association).

We have updated these within text descriptions throughout the results.

In a formal document strictly speaking, ‘knowlesi’ would be ‘P. knowlesi’.

We agree and have updated the manuscript to consistently refer to *P. knowlesi*, except for in the author summary, where we felt that knowlesi malaria (a standard term used in the malaria literature) would be more accessible for a lay readership.

‘multivariate analysis’ – I think you mean ‘multivariable analysis’. Multivariate has multiple outcomes whereas multivariable has multiple explanatory variables.

Thank you for this correction. We have changed this subheading and references to these results within the paper to state this is a multivariable analysis (Lines 271 and 387)

Journal Requirements:

1) Please upload all main figures as separate Figure files in .tif or .eps format. For more information about how to convert and format your figure files please see our guidelines:

<https://journals.plos.org/plosntds/s/figures>

This has been done.

2) We have noticed that you have uploaded Supporting Information files, but you have not included a list of legends. Please add a full list of legends for your Supporting Information files after the references list.

A full list of legends has been added after the references list.

3) We note that your Data Availability Statement is currently as follows: "All relevant data are within the manuscript and its Supporting Information files". Please confirm at this time whether or not your submission contains all raw data required to replicate the results of your study. Authors must share the “minimal data set” for their submission. PLOS defines the minimal data set to consist of the data required to replicate all study findings reported in the article, as well as related metadata and methods (https://journals.plos.org/plosone/s/data-availability#loc-minimal-data-set-definition).

For example, authors should submit the following data:

1) The values behind the means, standard deviations and other measures reported;

2) The values used to build graphs;

3) The points extracted from images for analysis..

Authors do not need to submit their entire data set if only a portion of the data was used in the reported study.

If your submission does not contain these data, please either upload them as Supporting Information files or deposit them to a stable, public repository and provide us with the relevant URLs, DOIs, or accession numbers. For a list of recommended repositories, please see https://journals.plos.org/plosone/s/recommended-repositories.

If there are ethical or legal restrictions on sharing a de-identified data set, please explain them in detail (e.g., data contain potentially sensitive information, data are owned by a third-party organization, etc.) and who has imposed them (e.g., an ethics committee). Please also provide contact information for a data access committee, ethics committee, or other institutional body to which data requests may be sent. If data are owned by a third party, please indicate how others may request data access.

De identified datasets and codes have been made available using a GitHub repository, with the following URL:

<https://github.com/PatrickAbraham1/Sabah-Knowlesi-COI>

4) Please amend your detailed Financial Disclosure statement. This is published with the article. It must therefore be completed in full sentences and contain the exact wording you wish to be published.

1) State the initials, alongside each funding source, of each author to receive each grant. For example: "This work was supported by the National Institutes of Health (####### to AM; ###### to CJ) and the National Science Foundation (###### to AM)."

2) State what role the funders took in the study. If the funders had no role in your study, please state: "The funders had no role in study design, data collection and analysis, decision to publish, or preparation of the manuscript.".

If you did not receive any funding for this study, please simply state: u201cThe authors received no specific funding for this work.u201d

We have amended this within the manuscript.

5) Please ensure that the funders and grant numbers match between the Financial Disclosure field and the Funding Information tab in your submission form. Note that the funders must be provided in the same order in both places as well. Currently, this grant number [1134989] provided by " the Australian Centre of Research Excellence in Malaria Elimination" is missing from the Funding Information tab.

Please indicate by return email the full and correct funding information for your study and confirm the order in which funding contributions should appear. Please be sure to indicate whether the funders played any role in the study design, data collection and analysis, decision to publish, or preparation of the manuscript.

This has been amended in the manuscript and stated via return email to the editor.

**Reviewers' Comments:**

Methods

Reviewer #2: The study presents a clear objective to use a household perspective in measuring and valuing out-of-pocket (OOP) expenses and productivity losses for episodes of malaria, disaggregated by malaria type. The study design is sound; however, I suggest adding more details on data collection methods (e.g., whether it involved clinic visits or phone interviews) and clarifying the patient selection criteria (e.g., was it every person presenting at the hospitals during the study period or a specific subset?).

We have added this in lines 115-119, including:

“A retrospective, cross-sectional cost survey was conducted across three district hospitals: Kudat, Kota Marudu, and Pitas, and one tertiary referral hospital in Kota Kinabalu. A pre-tested, structured, closed-ended questionnaire [24] was directly administered at clinics by trained research nurses to malaria patients or caregivers at around 28 days after hospital admission to estimate the costs of a single malaria episode.”

Additionally, further emphasis has been placed on the patient population in lines 105-108:

“Participants were included based upon an initial microscopic diagnosis of malaria, with final *Plasmodium* species determined by the laboratory PCR result. The patient population had an even spread across socio-economic quartiles, consistent across rural districts in Sabah[12].”

Comment 1: Please provide more information on how patients were contacted—whether through phone or in-person clinic visits—and clarify if participants received compensation for their time.

We appreciate this comment, which was also partly considered by the editor above. We have updated the study participants section accordingly. Lines 116-119 now reads:

“A pre-tested, structured, closed-ended questionnaire [24] was directly administered to all patients at clinics by trained research nurses to malaria patients or caregivers at around 28 days after hospital admission to estimate the costs of a single malaria episode [12]”.

Comments 2: Additionally, describe the questions used to collect cost data, especially regarding admissions or clinic costs. Did you ask a single question (e.g., "What did you pay in total?") or inquire about each cost component individually (e.g., “What did you pay for transport?” “What did you pay for food?”)? In the results (Line 212), you mention, "The majority of direct costs were related to hospital admission," which is unclear about whether these included only admission fees or also associated costs like food and supplies, suggesting a single-question approach.

We have added further details on cost data questions in lines 121-124:

“The out-of-pocket costs incurred by a household from a single malaria case were estimated using an ingredients-based approach, by individually questioning expenditure on travel, hospitalization, clinic visits, medications and lost productivity.”

Comment 3: Line 124: Please specify further what the two sources of monthly income are.

The sources of monthly income are in lines 132-134:

“Two alternate sources of monthly income (Malaysian Household Income Survey, and Household Inequities Survey [29, 30]) were also used…”

Comment 4: Line 128: Did you use the human capital approach? My understanding is that a consistent value for time lost (e.g., $X per day) is applied to all individuals, regardless of occupation. You note in the results (Line 23) that "Cost differences between sexes were primarily attributed to reported wage differences, with 65% (25/38) of females not reporting their wage losses.” This discrepancy is what the human capital approach aims to mitigate by assigning a standard value to time. Additionally, you mention (Line 127) including missed school hours; how did you value these, as they are not income-related?

This is a very interesting comment and something which is highly debated and discussed in health economic literature. Current literature lacks clarity over whether the “human capital” approach values time losses with a consistent wage, or whether “gross income” for individuals within studies should be used [1]. Given this uncertainty and debate, we do believe we have most accurately captured productivity losses using the human capital approach. In a recent systematic review, self-reported wages were shown to be a frequently used method for valuing lost time due to illness in low- and middle-income countries [1]; hence, we have used these wages as our base case. For the base case analysis, children did not have their time valued for these productivity losses; but in the scenario analyses (scenarios 3 and 5), we applied the standard wages to these children who missed schooling.

Comment 5: Line 162: Regarding the GLM, what was the rationale for choosing the specified variables? Did you conduct any model diagnostics to assess fit?

Thank you for this comment and we acknowledge that this should have been included in the original manuscript. We have addressed this in the methods, lines 165-167:

“Forward selection of potential clinical and demographic variables were used to fit the regression model based on Akaike information criterion (AIC).” Both AIC and BIC are included in the supplementary material where further details of the GLM characteristics can be found.

Reviewer #3: 4. I found the “study design and participants” section difficult to follow. Considering separate paragraphs for “study design” and “participants.”

We have changed the subheading of this paragraph to “Participants and study design” and have reorganised into separate paragraphs for participants and study design in lines 100-119.

Results

Reviewer #2: The analysis presented aligns well with the stated analysis plan, and the results are both clearly and comprehensively presented. The figures and tables provided are of high quality, making the findings accessible and easy to interpret.

We appreciate this positive comment.

Comment 6: The results section is very clear overall. To enhance clarity and alignment, I suggest structuring the presentation of costs similarly to the methods section. Presenting the direct out-of-pocket (OOP) expenditures first, followed by productivity losses, could provide a smoother transition into a combined "Household Cost of Malaria" summary.

Thank you for this suggestion. We have reordered this paragraph to reflect your suggestion (lines 221-234):

“Direct household costs equated to US$53.89 (SD=66.20) per person (41% of the total cost). The majority of direct costs were related to the hospital admission, although it is unclear whether these costs included direct admission costs or cost associated food and other supplies needed during an admission. Indirect household costs were the most significant contributor to the total cost burden (58%), with a mean total amount of US$75.84 (SD=69.67) for each household. When using self-reported wages (with mean wage imputed for adults who reported time off usual activities but not their income), the mean patient time cost was estimated to be US$53.60 (SD=58.77). The total caregiver time costs (including patients being looked after themselves and when alternative caregiving was needed to look after the patient’s dependents) were estimated to be US$22.24 (SD=28.40) for each malaria case. Only 3% (4/152) patients reported having “other costs” which were unspecified in the survey. These were <1% of the total costs, all were *P. knowlesi* cases, and three of these were reported by female patients. The mean total household cost per malaria episode was US$130.53 (SD=102.24) with a median of $96.11 (IQR=$70.73, $153.01) (Table 3).”

Comment 7: In Table 6, adding a per-month salary for each scenario would provide additional context for readers, allowing a clearer understanding of productivity loss within the scope of household economics.

Thank you for this comment, we have added this to Table 6.

Comment 8: The scenario analysis is an excellent addition to the paper and effectively highlights the assumptions driving productivity loss estimates. However, the paragraph presenting the scenario results could be made clearer. Consider reorganizing this information into a table to enhance readability—this could be incorporated into an existing table (e.g., Table 6) or presented in a new one.

We have updated this paragraph to improve clarity and included the wage estimate amounts in the footnotes of Table 6. We hope these changes improve the readability.

Conclusions

Reviewer #2: The conclusions are well-supported by the data presented, and the discussion section effectively summarizes the main findings, addressing key questions raised throughout the paper. The authors also highlight the study’s public health relevance, emphasizing the importance of understanding malaria’s economic impact on households.

We thank you for this praise.

Comment 9: Regarding the higher costs for P. vivax, did you collect data on the number of clinic visits for each family? This information could substantiate your claim in Line 350 that P. vivax cases involved more frequent clinic visits. Additionally, in explaining the cost difference, you mention that P. vivax predominantly affects younger patients. However, you also state that older patients tend to incur higher costs; it would be helpful to reconcile this point in the discussion.

This data was not collected and has been added as a limitation in lines 362-366-XX:

“Another factor which may contribute to higher costs is caregivers may be more likely to return their child for follow-up clinic services, and *P. vivax* requires a longer treatment duration of 14 days for the primaquine course, in contrast to other species that do not have dormant liver parasites to treat [21]. However, the survey did not collect data on the number of clinic visits for each patient.”

Comment 10: In Line 340, consider comparing your findings on catastrophic health expenditure to other studies. Discuss whether your findings align with, exceed, or fall below similar estimates in the literature, providing context to enhance the relevance of this result.

An excellent suggestion, we have now included previous estimates of CHE from malaria; however, no estimates from Asia were found. This has been updated and provided in lines 350-354:

“This is consistent with endemic settings in Africa, where catastrophic health expenditure due to malaria have ranged from 18-22%, however in vastly different health and economic contexts [2, 44-46]. No catastrophic health expenditure estimates for malarial illness have been estimated in Asian endemic settings.”

Comment 11: Line 398-400 appears to have missing words. Please review and clarify this sentence to ensure the intended meaning is conveyed.

This sentence now reads (Lines 416-418):

“Households with lower socio-economic status have been found to be at a higher risk of having malaria,[24] indicating the inequitable burden these households bear.”

Comment 12: Line 431: When mentioning that the study setting may have influenced cost estimates, please elaborate. Specify how the setting could have impacted the findings and in which direction (e.g., increased or decreased costs).

We have changed the wording to provide clarity (Lines 448-451),

“Additionally, recall bias and administering the survey as part of a research setting may have under- or overestimated the reported costs and resources spent by a household related to a single malaria episode [59].”

Comment 13: Line 442: Consider rephrasing this sentence to emphasize the utility of cost-of-illness data in informing cost-effectiveness analyses. Being explicit about this can clarify its relevance for policymakers and researchers.

We have added this to the conclusion. The sentence now reads (Lines 464-466):

“Given Malaysia's goal of malaria elimination, understanding the magnitude of household out-of-pocket costs and productivity losses is essential to inform policy and provide unit cost data for future cost-effectiveness analyses.”

Reviewer #3: 5. Because the species of malaria infection correlates with patient characteristics (e.g. people with P. knowlesi are more likely to be men), I’m left wondering whether it is the type of malaria infection or the characteristics of the patients driving these differences in cost. Are you able to disentangle that at all?

We have attempted to understand this through the Generalized Linear Model analysis. The GLM indicated that being male was not a significant variable associated with increased cost per malaria episode, however, being older was. Additionally, within the discussion we have stated:

“Without further data, it is challenging to speculate on other factors associated with higher total costs per malaria episode. However, when controlling for malaria severity, malaria from human-only *Plasmodium* species (predominantly influenced by higher *P. vivax* related costs) was associated with higher total costs (Table 5), which may be reflective of the longer duration of illness with *P. vivax* and *P. falciparum* compared with non-severe *P. knowlesi* (Table 2).”

Editorial and Data Presentation Modifications

Reviewer #1: Reader may benefit from having more visual presentation of data/results - at the moment the results are primarily detailed in tables.

While we generally agree that more visual presentation of results would be preferable, we were not able to think of additional figures which would aid in the messaging and novelty of our research. If there are any specific suggestions of additional figures, we are very open to including these in future revisions. As stated in the conclusion, much of the value of this research are the unit costs which may be used in further cost-effectiveness studies. Hence, the unit costs presented in Tables 3-6 are essential. We have an additional figure of the scenario analyses by age categories in the supplementary material.

Reviewer #2: I’ve provided detailed comments for the methods, results, and discussion/conclusions sections, including editorial suggestions and recommendations for data presentation. In addition, I have some suggestions below for the introduction, which is overall well-written and provides a strong foundation for the study.

Comment 14: On Line 51-52, it may be clearer to report the number of malaria cases directly instead of using the term “public health notifications,” which could be confusing for readers unfamiliar with the phrasing.

Thank you for this comment, we have amended this line to now state

“*P. knowlesi* is now the major cause of malaria in Malaysia, with malaria case notifications increasing to 3000-4000 cases per year in 2017 and remaining at this level currently [5-7].”

Comment 15: Line 54: Please include the corresponding percentage for P. falciparum cases. Additionally, the sentence structure suggests that all patients presenting at a health facility have severe disease, which is not consistent with the information provided later. Clarifying this here would avoid any misinterpretation.

Thank you for this comment, we acknowledge the wording as the cause of confusion. We have updated this to:

“*P. knowlesi* infection also carries a high risk of severe disease, with 6-9% of symptomatic cases presenting to health facilities in East Malaysia classified as severe malaria, comparable with previous risk for falciparum malaria (5-11%) in sympatric areas [5, 8-10].”

Comment 16: Line 78-80: Consider rephrasing to: “However, households still incur out-of-pocket costs for travel, medicines, and healthcare visits, which, to our knowledge, have not been previously estimated.” This phrasing improves clarity and flow.

We have updated based on this suggestion in Lines 78-80.

Comment 17: Line 47: You mention that there have been no indigenous cases of P. falciparum or P. vivax in five years, but several cases were observed in this study. If this discrepancy is due to the study period (2013–2016), please clarify in the discussion to address potential reader confusion.

We have stated this in the discussion, lines 460-462:

“Lastly, the data in this study is dated, with no indigenous cases of *P. falciparum* and *P. vivax* present in the area, which limits the applicability of these findings, however there are still imported cases.”

Summary and General Comments

Reviewer #1: General comments: A thorough and thoughtful costing exercise. I appreciated the transparency of the methods and clearly the authors spent time investigating the quirks of their data (e.g. the anecdote to explain the cost differences between males/females). I think the methods and results are all strong and comprehensive. Though the conclusions could be strengthened to drive home the importance of the findings to reinforce statements like "Our research also highlights the opportunity cost of not developing novel public health interventions to control P. knowlesi transmission." This would be strengthened by a simple multiplication of the patient costs per case and an estimate of the number of knowlesi cases per year in Sabah. Presumably these costs will be used (or would be useful) in cost-effectiveness studies in Sabah/Malaysia of interventions from a societal perspective and more fully capture the impact of these interventions - would be good to state this.

We have amended the conclusion to reflect the usefulness of this study for future cost-effectiveness analyses in Malaysia, and how they findings could be used to help inform policy, lines 464-466:

“Given Malaysia's goal of malaria elimination, understanding the magnitude of household out-of-pocket costs and productivity losses is essential to inform policy, and guide novel interventions and enable future cost-effectiveness analyses.”

There are several reasons why we did not multiply the cost per case by the number of cases:

1. The malaria endemicity in Sabah has changed considerably since the study data was collected.
2. Notified cases of knowlesi malaria are likely to be under-reported in these settings, so by extrapolating there is a chance we would be underrepresenting the true cost of illness.
3. Given the uncertainty in the scenario analyses we have presented, we believe that extrapolating would lead to greater uncertainty than we would be comfortable with publishing.

Main comments:

i) The data were collected between 2013 and 2016, this is quite a while ago. I think there needs to be some acknowledgement of this and brief discussion of the generalisability to the situation today in Malaysia.

We have stated this in the discussion, lines 460-462:

“Lastly, the data in this study is dated, with no indigenous cases of P. falciparum and P. vivax present in the area, which limits the applicability of these findings, however there are still imported cases.”

ii) Would be useful to understand more about the economic situation of Sabah, how does it sit comparatively in terms of wealth to other parts of Malaysia?

This is a valid point and has until this point been left out of our analysis. Sabah has one of the lowest GDP per capita of all Malaysian States. Due to this, it is politically sensitive to directly address this in the introduction. We have provided a reference to the original surveillance study and explained the general SES of the patient population within the state. in lines 107-108:

“The patient population had an even spread across socio-economic quartiles, consistent across rural districts in Sabah [12].”

We also have alluded to the SES of this patient population in the Discussion, lines 398-409:

“Despite government health services being provided at no charge for malaria patients (e.g. usual hospital admission fee, routine antimalarial medicine costs, laboratory testing), out-of-pocket costs for households still have the potential to impact those experiencing socio-economic disadvantage disproportionally. Whilst this study did not directly capture socioeconomic disadvantage, understanding occupational status and district of residence may provide insight into subpopulations experiencing disadvantage. Irrespective of the method used to estimate wages and the health expenditure threshold chosen, a large proportion of malaria patients exceeded the catastrophic health expenditure threshold for mean direct out-of-pocket costs. It is conceivable that for many lower-income households, dealing with the impact of this type of health expenditure may necessitate either deferring initial or subsequent care, particularly considering the self-reported income from participants in the study was far less than the estimated average incomes from the region.”

iii) The authors stated that the analysis is from a household perspective yet household earnings were not measured, only the patient's. The authors acknowledged the catastrophic health expenditure calculation is performed only using the proportion of the individuals earnings. However, other members of the households costs were included (e.g. caring responsibilities etc). So the costs are accrued from the entire household yet divided by the individuals earnings? If I have understood that correctly then this would likely overestimate the cases of catastrophic health expenditure. Is it possible to use alternative estimates of average household, rather than individuals, incomes in Sabah as the denominator? At the very least this should be explored as a scenario analysis. Or if just the individual's earnings are being used as the threshold, then perhaps only the individuals costs should be used as the numerator (though the perspective would then be the patient's perspective rather than households).

We very much appreciate this comment and consideration, and we have spent a lot of time and research discussing the best approach for this. We considered using a monthly income of double the reported (representing two incomes), however decided against this. Most of our sample (75%) were males, and the female workforce participation in Sabah, Malaysia is less than 50%. Therefore, this would likely over-estimate household monthly income for this population. Accordingly, we concluded that using the actual reported income was the soundest methodology, although this may have led to over-estimation of catastrophic healthcare expenditure. Given we have used several sources of income, and two thresholds (10 and 25%) to estimate catastrophic healthcare expenditure, additional adjustments for household income may lead to greater uncertainty of these. We have acknowledged this as a limitation in lines 452-454:

“Additionally, no questions were directly asked about total household income, thus if household have multiple income sources, they have been unintentionally excluded in catastrophic health expenditure calculation.”

iv) I appreciate data on socioeconomic status were not collected, however it would be interesting if the authors can infer something about SES from the data they do have to help describe the patients who are presenting. E.g. from the reported earnings data vs Sabah/Malaysian average wages.

Thank you for this comment. This has now been stated in lines 107-108 in the methods,

“The patient population had an even spread across socio-economic quartiles, consistent across rural districts in Sabah.”

The discussion in lines 406-409 now states

“It is conceivable that for many lower-income households, dealing with the impact of this type of health expenditure may necessitate either deferring initial or subsequent care, particularly considering the self-reported income from participants in the study was far less than the estimated average incomes from the region.”

v) This study will probably be published in 2025, yet the reported costs are in 2022 USD. Could the authors inflate these costs to 2024 values?

We have updated the costs to 2023 USD. Exchange rates and GDP deflators to update costs to 2024 will not be available until mid-2025.

vi) The introduction states malaria testing and treatment is free under the UHC program in Malaysia yet there are direct medical costs included in the results - this appears to be a contradiction to me and seems an important point to clarify, are these copayments under the insurance scheme? Are these patients for whatever reason ineligible from insurance coverage (e.g. due to nationality - was that captured?). The authors state "it is unclear whether these costs included direct admission costs or cost associated food (sic) and other supplies needed during an admission" - My sense is it seems a bit high for just food costs alone. It is also entirely possible that hospitals levy fees despite policies in place but it would be good to clarify the case here if possible. The combined possible direct medical costs make up $45.76 (33%) of the total and may be avoidable by policy/enforcement of policy whereas to some extent productivity losses can only be reduced by reducing the incidence of malaria

Unfortunately, no further details were collected about these costs. While it is possible that hospitals charge indirect fees for care or to improve the care provided to patients, this may be politically sensitive to speculate on in our paper without further data.

Minor comments:

i) Page 19, line 306: "When using the mean monthly self-reported income value of US$168, direct health expenditure for a single malaria episode accounted for 27% of monthly income." The $168 is different to the value reported earlier in the paper ($156) - is this because this is the inflated value? Needs clarification.

Thank you for pointing this out, this was an error, and was updated to $156, which is now $158.76 in 2023 USD.

ii) Page 22, line 399: "indicating the inequitable these households bear." Sentence needs adjusting.

This has been updated to

"indicating the inequitable burden these households bear."

iii) Page 24, line 436: "they have been missed in catastrophic health expenditure calculation" Sentence needs adjusting.

We have updated the wording to:

“Additionally, no questions were directly asked about total household income; thus, if household have multiple income sources, they have been unintentionally excluded in catastrophic health expenditure calculation”

Reviewer #2: The manuscript, “The economic burden of zoonotic Plasmodium knowlesi malaria on households in Sabah, Malaysia compared to malaria from human-only Plasmodium species,” provides a novel and valuable contribution to understanding the financial impact of malaria at the household level, particularly by differentiating costs by malaria parasite type. This study fills an important gap in the literature by highlighting the economic burden of malaria in Malaysia and has potential implications for both public health and economic policy.

The paper is clearly written, logically structured, and effectively guides the reader through its key findings and implications. The finding that P. vivax incurs higher costs than other malaria types may seem unexpected, but the authors do an excellent job of presenting plausible explanations for this in the discussion, addressing factors such as age distribution and frequency of clinic visits among patients with P. vivax. Their discussion supports the robustness of the study’s conclusions.

The methodological execution is rigorous, and the inclusion of both direct and indirect household costs is commendable. However, two areas could benefit from further clarification. First, the scenario analyses, while methodologically sound, could be made more interpretable for the reader by enhancing the presentation in the results section, possibly through more detailed description or an additional table summarizing the scenarios.

Thank you for these kind comments. As addressed above, the scenario analysis results section has been extensively revised to improve readability.

Second, more explanation on the variable selection in the modeling process would strengthen the paper. Specifically, clarifying whether the model was theory-based, derived from backward selection etc. would provide insight into the modeling rationale and improve transparency. Also the authors do not mention any regression model diagnostics are used to assessed the validity and reliability of a model's results.

We acknowledge that this should have been included in the original manuscript. We have addressed this in the methods, lines 165-167:

“Forward selection of potential clinical and demographic variables was used to fit the regression model based on Akaike information criterion (AIC).”

Both AIC and BIC are included in the supplementary material where further details of the GLM characteristics can be found.

These minor revisions would enhance the clarity and interpretability of an already strong manuscript. Congratulations to the authors for their thorough work and significant contributions to understanding malaria’s economic impact on households. I look forward to seeing these revisions in the final version.

Thank you.

Reviewer #3: 1. In my view, the most interesting/important finding was that most patients are incurring catastrophic health expenditures regardless (if I’m understanding it correctly) of the species of malaria infection and despite the country’s policy of covering malaria treatment expenses. I don’t know the Malaysian context well, but I suspect that if malaria elimination is the goal, there is already motivation to address P. knowlesi. The fact that P. knowlesi costs are not very different from other malaria costs may just reinforce the status quo. It could be a more impactful paper if it has a health financing message rather than a malaria control message.

Given the malaria endemicity in Malaysia has changed since the data in this study was collected, we believe the current framing is the most useful for both Malaysia. To our knowledge, this is one of the first studies to compare the cost of different species of malaria in one location. Accordingly, we have updated the title to:

“Higher economic burden of *P. vivax* compared to *P. falciparum* and zoonotic *P. knowlesi* on households in Sabah, Malaysia.”

We hope you agree that this better reflects the results and messaging of the study.

2. If you opt to keep the current framing, the Background section needs to make a stronger case as to why comparing the costs of different species of malaria is important, and better justify the need for the subgroup analysis. Including hypotheses would be helpful. What cost differences did you expect there to be, and why? Some of this content could be relocated from the Discussion, which is quite long. There is also some contextualization in the Results (lines 256-7), which should be moved elsewhere.

Thank you for this suggestion. We do not feel comfortable with retrospectively stating a hypothesis for this study. However, we state in the introduction

“*P. knowlesi* infection also carries a high risk of severe disease, with 6-9% of symptomatic cases presenting to health facilities in East Malaysia classified as severe malaria, comparable with previous risk for falciparum malaria (5-11%) in sympatric areas [5, 8-10].”

We have added some extra detail around cost comparison of species in the introduction, lines 82-86:

“No previous studies have estimated the cost of *P. knowlesi* infections, nor where many species of malaria are found within the same area, however *P. vivax* and *P. falciparum* have shown to have similar costs [24]. Additionally, *P. malariae* commonly presents very low parasitemia and mild disease severity [25].

We have removed the content from lines 256-7 to the introduction as you have suggested and believe this aids the narrative which needed to be presented more clearly about species cost comparison.

We have attempted to be more succinct in the discussion.

3. This is a small concern, but under “Ethics,” it would be better to list all of the IRB’s than to say the IRB’s included XYZ.

While we are happy to comply with this request, we are unsure about how to do so. Could the editor please clarify how to address this?

Selected reference

1.	Setiawan E, Cassidy-Seyoum SA, Thriemer K, Carvalho N, Devine A. A Systematic Review of Methods for Estimating Productivity Losses due to Illness or Caregiving in Low-and Middle-Income Countries. PharmacoEconomics. 2024:1-13.
