# Supplementary material for: Household costs associated with zoonotic Plasmodium knowlesi, P. falciparum, P. vivax and P. malariae infections in Sabah, Malaysia
Source: PLoS Negl Trop Dis. 2025 Apr 4;19(4):e0012180. doi: 10.1371/journal.pntd.0012180 (PMC11970689; doi:10.1371/journal.pntd.0012180)
Supplement: Attachment — Submitted filename: Response to Reviewers_2.docx [file pntd.0012180.s005.docx]

**Additional Editor Comments:**

Please respond to the small comment from Reviewer 2

This has been addressed in L117 and stated below.

Some of the responses to comments have introduced some confusion:

- Reviewer 3 had commented that the paper may have a stronger message if the paper has a health financing message rather than a malaria control message. The authors responded by changing the title to compare P vivax to the other malarias (rather than the original version comparing P knowlesi to the other malarias) on the basis that P vivax tends to have the higher costs. This does not address the comment directly and has the unfortunate side-effect that the title is now inconsistent and jars with the introduction, results and discussion which focus on the comparison of P knowlesi to P vivax and P falciparum.
Please change either the title or the text so that they are consistent, and consider whether the message should include a health financing angle.

Given that malaria endemicity has changed dramatically since these data were collected, we still do not feel that we can appropriately write this manuscript as a health financing paper. We do note that there are many financing elements presented in this article, including the overall message that despite “free” malaria care, the current financing structure may not be adequate in fiscally supporting malaria patients. However, we now agree that there was a lack of consistency in messaging throughout the manuscript following the extensive revisions in the last round, including the title of the manuscript.

While we thought it would be best for our title to reflect the main findings from our study (now revised to state that we’re reporting costs on the four species of malaria present), the story of the paper focuses more on knowlesi malaria for two reasons.

The first is because of its relatively new emergence as a public health issue. The second is because this is the first time that the costs associated with an episode have not been presented. (While this is also true for malariae cases, the sample size was much smaller and it is currently a lesser public health threat.) We have made substantial revisions to the title, abstract, introduction, and discussion to try to do a better job of interweaving these stories. We hope that these revisions now align more closely with the presented results.

- Forward selection of variables for the GLM
I could find one value of AIC in the legend for table S7. However it is not clear how forward selection was carried out - was there a cut-off for the AIC change used for selecting variables? There are some non-significant variables included in the table and in the GLM. Forward selection is not necessary or recommended, simply considering likely confounders from prior knowledge would be fine. However if forward selection is mentioned, it ought to be clear what the criterion for inclusion was.

Thank you for this feedback.

When we initially performed the forward selection, AIC reduced with each variable added. However, the confounders were originally chosen from prior knowledge and consultation with the clinicians and economists within the authorship team. We have updated this line to now read (L164) “Likely confounders were based on prior knowledge, and plausibility of variables which may have impacted costs.” We have chosen to keep the AIC in Table S7 as a descriptive statistic.

- A previous comment on the sample size was been responded to but rather indirectly. Please check that it is clearly stated that the sample size in this study was small for P vivax, P malariae and P falciparum.

This has now been directly stated in the limitations (Line 432): “Case numbers were also low for *P. vivax*, *P. falciparum* and *P. malariae* infections.”

- L273 - Lower bound of the CI is $-19 but in the table it is -119. The very wide CI in the table for the GLM suggest that the GLM is squeezing the small sample size rather hard or perhaps that the gamma distribution does not fit so well. It would be useful to check the statistics. Some of the CI bounds are not obviously plausible.

The typo in the table has now been corrected as was correctly stated on L271 as -$19, which is the correct CI from the GLM. We do agree the large confidence interval for severe malaria is likely due to the small sample size of severe cases. Due to the skewness of our cost data (and the nature of most cost data), gamma distributions are recommended for these types of data. Upon review, we explored various model fits with Poisson, Gaussian and various binomial (logit and probit link) and the gamma distribution (log link) was the best fit from all model fit statistics.

- Standard deviations are included in the abstract for the overall costs however they are also needed for the species-specific costs.

This has now been done, with standard deviations stated for each species in the abstract and main text.

- L254 p=<0.001 typo (it should be p<0.001)

This has now been corrected to p<0.001.

The reasons for the tendency for higher costs of P vivax are not consistent - in the discussion the different reasons are nicely explained, however in the abstract the message is different and one reason only is implicated.

We have more strongly aligned the abstract to the discussion regarding this. L22-23 now reads “The higher *P. vivax* costs may have been due to a younger age profile, and longer treatment for the required liver stage treatment for these patients.”

 Reviewer #2: The authors have adequately answered all of my questions and comments from the first round of review. **One small suggestion - please report if participants received compensation for their time.**

This has now been stated in line 117. “No payments were made to participants.”

**Journal Requirements:**

Please ensure that the funders and grant numbers match between the Financial Disclosure field and the Funding Information tab in your submission form. Note that the funders must be provided in the same order in both places as well.

I have attempted to do this, but please advise on further details if something is still incorrect.
